# Coupling feeding activity, growth rates and molecular data shows dietetic needs of *Ciona robusta* (Ascidiacea, Phlebobranchia) in automatic culture plants

**DOI:** 10.1038/s41598-020-68031-0

**Published:** 2020-07-09

**Authors:** Valerio Zupo, Sebastiano Scibelli, Mirko Mutalipassi, Nadia Ruocco, Francesco Esposito, Alberto Macina, Gianluca Polese, Anna Di Cosmo, Maria Costantini

**Affiliations:** 1 Department of Marine Biotechnology, Stazione Zoologica Anton Dohrn, Villa Comunale, 80121 Naples, Italy; 2 Department of Biology, University Federico II of Naples, Monte Sant’Angelo, 80126 Naples, Italy; 3 Department of Research Infrastructures for Marine Biological Resources, Marine Organism Core Facility, Stazione Zoologica Anton Dohrn, Villa Comunale, 80121 Naples, Italy

**Keywords:** Developmental biology, Ecology, Zoology, Biological models, Gene expression analysis, Biological techniques, Microscopy, Transmission light microscopy

## Abstract

The sea squirt *Ciona robusta* is a model organism characterized by a transparent body, exhibiting peculiar physiologic and evolutionary characters. In vitro fertilization and breeding of sea squirts is possible, in order to preserve consistent genetic pools. However, some aspects of its biology, as the feeding efficiency according to diet quantity and quality, are still scarcely known. Here we test the effects of three experimental diets on survival and growth, to detect physiological and molecular responses to various types of alimentary suspended particles and the effects of feed concentrations. We also aimed at determining rearing conditions able to limit handling operations, save artificial seawater and control water pollution. Molecular analyses of growth-related genes were performed to detect stressful effects due to feed quality and quantity. A strong effect of doses was highlighted, but water pollution may represent a major concern. A compound diet containing both live algae and non-live particles of a correct size is indispensable to assure development, low stress and high survival rates. Overall, our findings suggest protocols for an easier rearing of *Ciona robusta* in the laboratory, increasing the potentialities of these organisms as models for research.

## Introduction

The solitary ascidian *Ciona robusta,* exhibiting an almost transparent body, is characterized by a wide geographical distribution^1^. It is a euryhaline and eurytherm species^2,3^ living in areas of high turbidity and in coastal lagoons^4–6^. Several descriptions of *C. robusta* are available from various locations around the world, along with studies on its taxonomy and the main synonymies^7^. This organism may be a dominant member of benthic communities along European coasts^8^, reaching high densities (as high as 2,000 individuals per m^2^ in polluted communities^9,10^), especially in colder seasons^6^. Its wide environmental adaptability permits to live in polluted harbours, as well as in dense seagrass (*Posidonia oceanica*) meadows^11^. It is sensitive to various types of pollution, especially in warmer seasons^12^, when its population naturally decreases^13^ but information about the factors influencing winter blooms is still incomplete^5,14^. However, the alternation of demographic explosions and consistent numerical reductions is typical of other species of solitary ascidians^15,16^, representing an aspect of their life cycle, still poorly known^17^, that is considered age and density-independent^18–20^.

Its semi-transparent body not only permits the observation of the internal anatomy, but also to follow the embryo development (after dechorionation, i.e., removal of the follicular cells covering its eggs). The body colour at the distal end of siphons is the main external character distinguishing sister species within its species complex^21^. It is a hermaphroditic broadcast spawner, but cannot self-fertilize^22^. *C. robusta* is mostly self-sterile and it has been used for studies on the mechanism of self-incompatibility^23^. Its genome has been fully sequenced and annotated^24^ and various mechanisms, including the biology of endostyle^25^ (precursor of a human thyroid), the fertilization process^26^ and the ability to react to various allelochemicals^8,27^, have been investigated. It is an easy-to-breed organism that can be reared in relatively small tanks^26^. For this reason, it is considered a model organism for scientific research^28^ especially for genetic^29^, genomic, regeneration^30^, and various medical studies^31^. In fact, genetic screens, germline transgenesis, electroporation of plasmid DNA and microinjection of morpholinos are all routinely employed on this species and complemented by targeted mutagenesis, homologous recombination, and RNAi^30^. In addition, many lines of transgenic animals expressing fluorescent markers in a wide array of patterns, under the control of endogenous or exogenous promoters, have been generated and are available for medical studies^32^.

However, *C. robusta* is becoming an endangered resource in some areas^33^, due to global and local environmental changes impacting its survival. Despite its abundance and the plethora of scientific investigations on its ecology and molecular biology, our knowledge of its feeding physiology is still incomplete^34,35^, and this limits our possibilities for culturing genetically consistent populations for scientific purposes. In fact, improvements of culture methods for marine invertebrates in closed system are prerequisites for a generalized use of transgenic lines^26^.

*Ciona robusta* is a suspension filter-feeder that removes particles from the water column^36^. Food particles are further conveyed into the intestine, which bends upwards, from the base of the stomach, to join the rectum and opens into the wall of the atrial cavity, expelling faecal pellets through the atrial siphon^37^. It produces a mucous lattice^38^ that captures the collected food particles forming a string, further transported to the oesophagus and the stomach, which is located beneath the gill basket, moving from the languets of the dorsal lamina^39^. Various experiments demonstrated a lower retention limit of 1–2 μm for captured particles, and that such small particles as bacteria can also be captured^40^. Most laboratory studies on the filtration rate and size of ingested particles were performed using uniform-size unicellular algal cultures, not reflecting the actual size variability of particles recorded in nature^34^. However, several reports on inefficient retention rates of larger particles were based on comparisons of pumping and clearance rates^34^, rather than direct measurement of retention efficiency. In our case, we tested the efficiency on growth rates and mortality rates of such different particles with average size ranging from yeast cells (3–40 µm) to larger algae contained in the Shellfish diet and Algamac (300–600 µm).

Overall, filtration rates depend on the size of *C. robusta* but feeding rates considerably intensify with increasing concentration of algal cells and drop at the highest concentrations (20,000 cells mL^−1^)^41^. Feeding studies on marine invertebrates demonstrated the importance of dietetic regimes (both their composition and their particle size distribution) to promote the growth of post-larvae and adults^42–44^.

*Ciona robusta* has been maintained in scientific laboratories since the last century, kept on a diet of diatoms, so demonstrating the feasibility of long-term culture for such model invertebrates. In addition, some laboratories set ascidian culture facilities close to the sea, facilitating large-scale productions. In this case, seawater naturally containing plankton was pumped into the tanks^26^. However, several points are of critical importance in closed systems for establishing healthy populations in continuous culture, while reducing costs of production. Food is a key element for the culture^26^ of *Ciona* and it must be cheap and easy to obtain and to prepare; in addition, it must provide indispensable compounds for its growth and reproduction, assuring low levels of water pollution. Previous authors considered a live natural diet as the most appropriate, especially for juveniles, due to its small particle size and low levels of pollution. This may represent a major advantage over artificial foods, particularly when seawater availability is limited. However, this method imposes the cultivation of live algae, considered as an expensive practice in aquaculture^42^. Moreover, periodical contamination requires efforts to re-establish mother cultures. To avoid these problems, some authors applied simple artificial diets based on commercially available mixtures of dry or suspended microalgae. The results in terms of growth and health of mature individuals, however, have never been directly compared with those made possible by natural diets^36^. In addition, filter-feeders have been successfully cultured simply by improving bacterial proliferation, using simple substrates and yeasts, but this strategy was never attempted for culturing *C. robusta*^26^. Here we propose a direct comparison of results made possible by the adoption of three culture strategies: (a) easy administration of a conserved algal complex commercially available; (b) adoption of a complex dietetic regime containing both live algae and organic suspended particles; (c) administration of a simple feed made of dry milk and dried algae, to take advantage of both plant and animal feeding principles in their simplest formulation. Experiments were performed by employing an automatic culture device, at two levels of particle concentration, in order to provide for the first time a comprehensive and direct comparison of growth rates promoted by different strategies and identify the best feeding efficiencies according to production costs. The efficiency of diets was evaluated by comparing growth and survival rates, but we also used qPCR to detect dose-dependent stressful conditions and test the hypothesis that dietetic regimes might affect the expression levels of genes involved in developmental processes of sea squirts.

## Results

### General conditions of tanks

**Figure 1:**
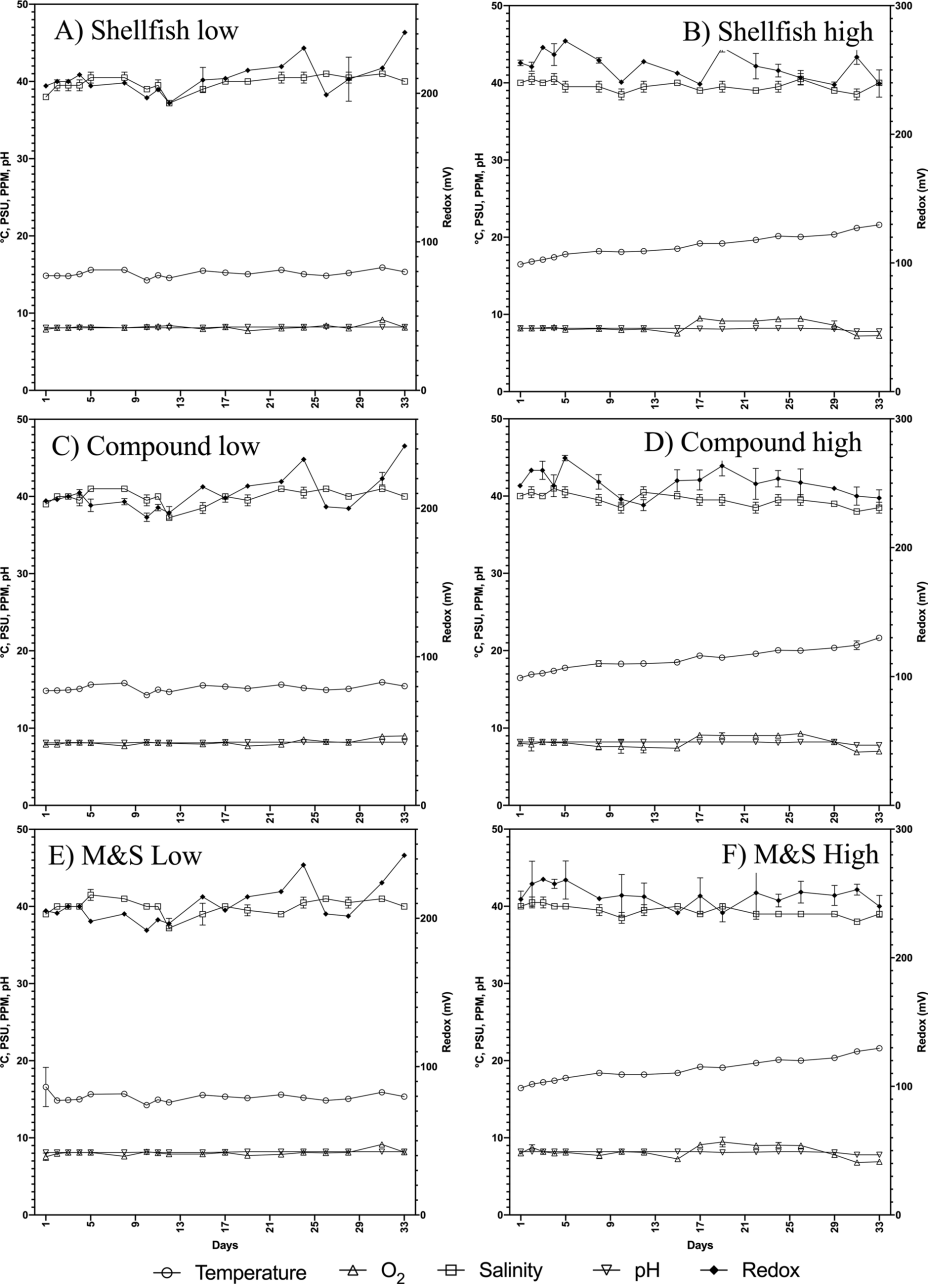
Main abiotic features of water measured in tanks managed at low doses of feeds (**A**,**C**,**E**) and high density of feeds (**B**,**D**,**F**) according to the feeding treatments reported in methods. (**A**,**B**) Shellfish; (**C**,**D**) Compound; (**E**,**F**) Milk&Spirulina (indicated as M&F). Figure produced using GraphPad Prism 8.0.0 for Mac, GraphPad Software, San Diego, California USA; http://www.graphpad.com.

Chemical and physical parameters of water were kept within the optimal ranges known for the survival of *C. robusta* during our experimental trials (Fig. 1). Average temperature was 15.2 °C ± 0.44; pH was maintained at 8.1 ± 0.05; dissolved Oxygen was kept at saturation values, with mean values of 8.3 mg/L ± 0.36; salinity was on average 39.9 PSU ± 0.97. Redox potential ranged from 192.5 to 243.5 mV, also according to the quality of impelled seawater (Fig. 1), but the range of variation of Redox potential in the tanks managed at low concentration of feeds (Fig. 1A,C,E) was higher than in tanks at high concentration of feeds.

**Figure 2:**
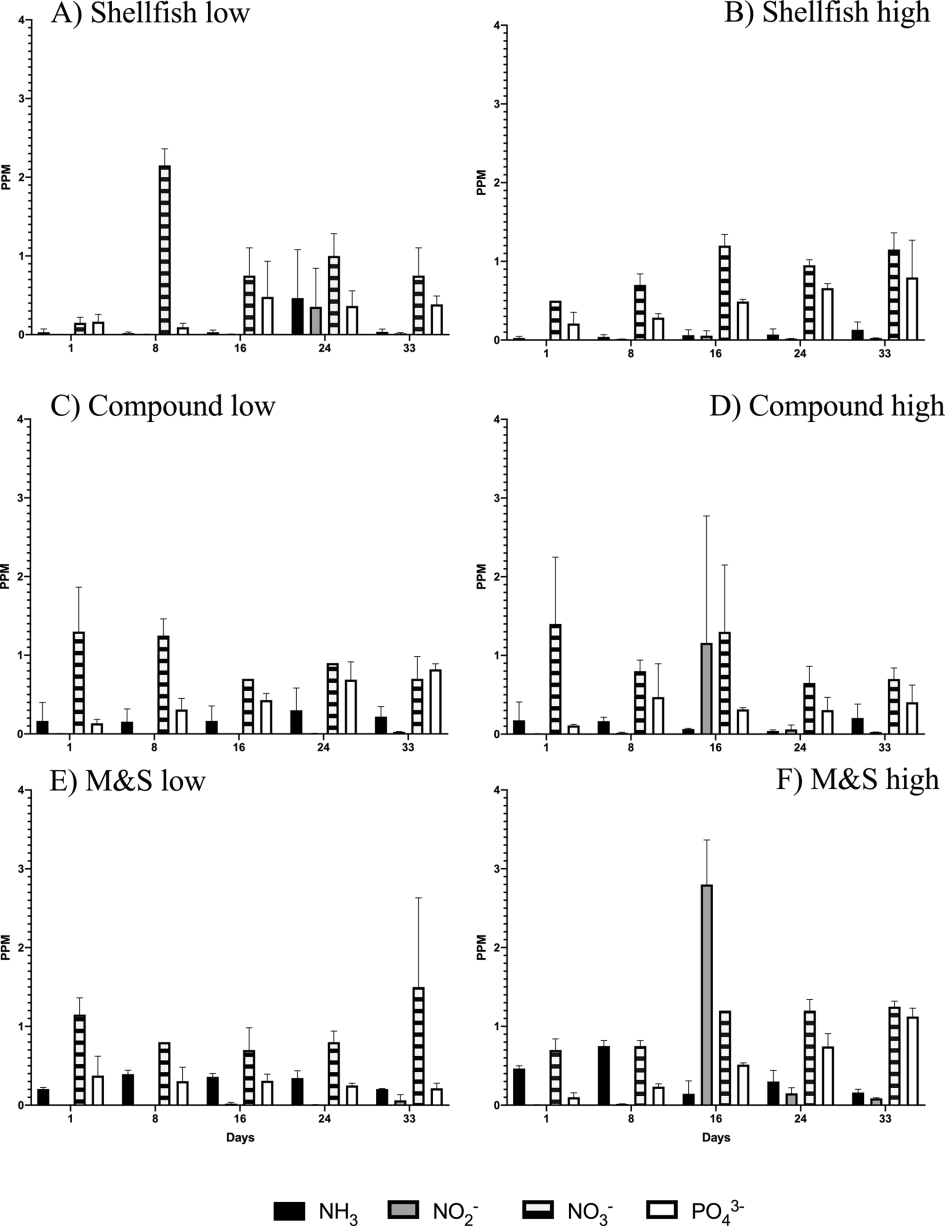
Levels of the main nutrients dissolved in the seawater according to the dietetic regimes reported in methods. (**A**,**B**) Shellfish at low and high doses, respectively; (**C**,**D**) Compound feeds at low and high doses, respectively; (**E**,**F**) Milk&Spirulina at low and high doses, respectively. Figure produced using GraphPad Prism 8.0.0 for Mac, GraphPad Software, San Diego, California USA; http://www.graphpad.com.

Nutrient concentrations in most treatments were kept almost constant by the automatic culture system, up to the end of the experiment (Table 1), although higher concentrations of nitrites and ammonia characterized the treatment Milk&Spirulina (further indicated as M&S; Fig. 2E,F). Nitrate concentration was on average 0.76 ± 0.17 mg/L (Fig. 2C,D) and exhibited maximum values in the treatment Shellfish at both doses (Fig. 2A,B); phosphate concentration was on average 1.03 ± 0.74 mg/L and exhibited higher values in treatments Shellfish and M&S at high concentration of feeds (Fig. 2B,F). On the whole, chemical and physical conditions of tanks were similar and most differences among treatments were not significant (Mann–Whitney test, p > 0.05).

**Table 1:** Main nutrients and chemical descriptors of water quality as measured in the replicate tanks at the end of the experiment. Average values and their standard deviations for three dietetic regimes at low and high concentration of feed particles. The names of treatments are referred to the compositions of feeds indicated in Table 3.

	Shellfish		M&S		Compound	
	Low	High	Low	High	Low	High
ppm NH_3_	0.035 ± 0.04	0.130 ± 0.09	0.205 ± 0.01	0.160 ± 0.04	0.220 ± 0.13	0.205 ± 0.18
ppm NO_2_	0.016 ± 0.01	0.0245 ± 0.00	0.061 ± 0.07	0.0865 ± 0.01	0.025 ± 0.01	0.025 ± 0.00
ppm NO_3_	0.75 ± 0.35	1.15 ± 0.21	1.50 ± 1.13	1.25 ± 0.07	0.70 ± 0.28	0.70 ± 0.14
ppm PO_4_	0.385 ± 0.11	0.795 ± 0.47	0.215 ± 0.06	1.125 ± 0.11	0.82 ± 0.07	0.405 ± 0.22
T°C	15.3 ± 0.07	21.6 ± 0.00	15.3 ± 0.07	21.6 ± 0.00	15.4 ± 0.21	21.6 ± 0.20
Salinity PSU	40 ± 0.01	38 ± 0.70	40 ± 0.01	39 ± 0.01	40 ± 0.01	38 ± 0.71
ppm O_2_	8.15 ± 0.07	7.25 ± 0.07	9 ± 0.01	6.90 ± 0.01	8.15 ± 0.07	7 ± 0.14
pH	8.2 ± 0.00	7.8 ± 0.00	8.2 ± 0.00	7.8 ± 0.00	8.2 ± 0.00	7.8 ± 0.00
mV Redox	241 ± 1.41	327 ± 53.00	242 ± 0.01	320 ± 28.30	242 ± 0.71	326 ± 96.20

### Survival rates

**Figure 3:**
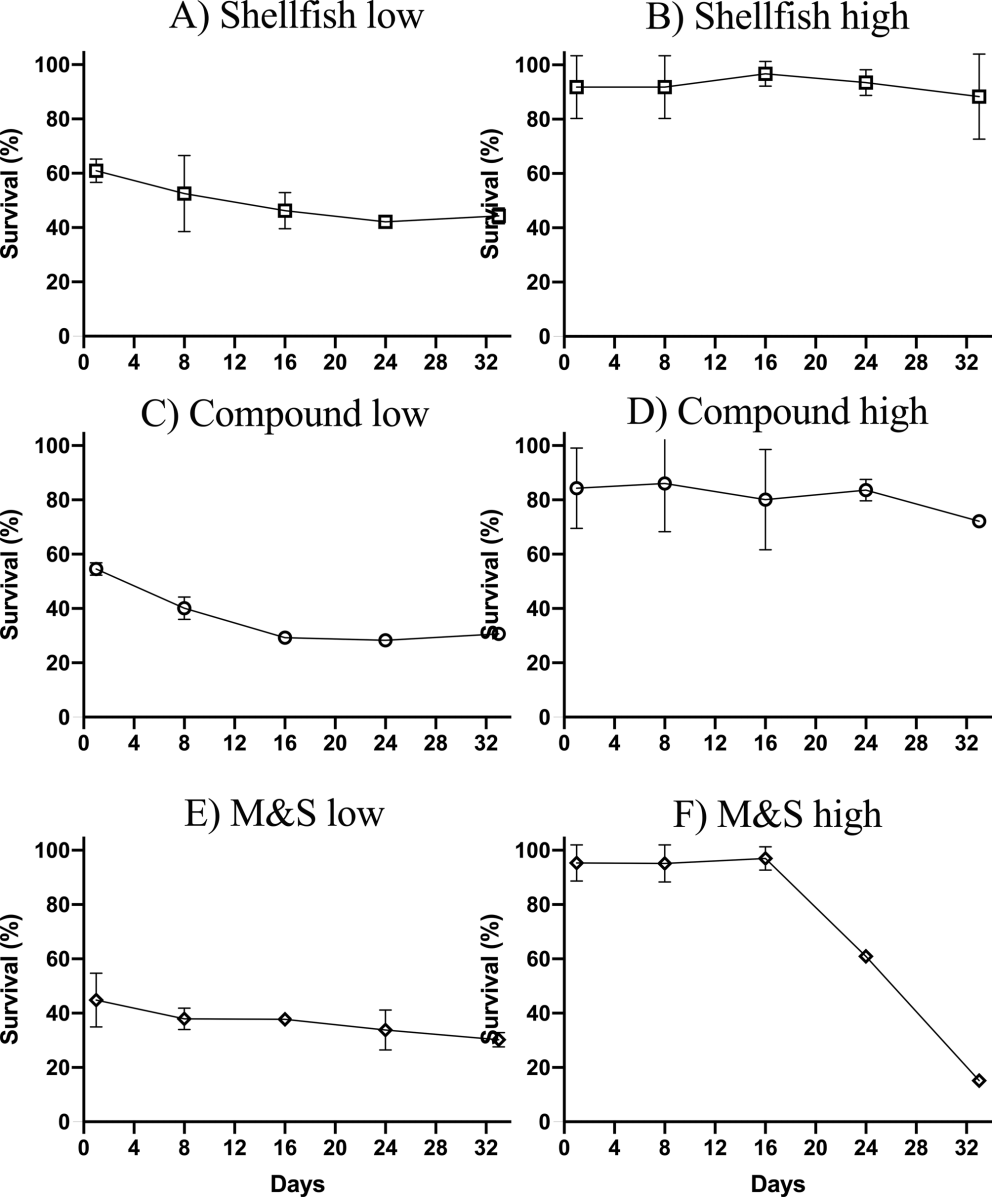
(**A**,**C**,**E**) Survival rates obtained in three dietetic regimes (Shellfish, Compound, Milk&Spirulina feeds, respectively) at low doses; (**B**,**D**,**F**) survival rates obtained in three dietetic regimes (Shellfish, Compound, Milk&Spirulina feeds, respectively) at high doses. Figure produced using GraphPad Prism 8.0.0 for Mac, GraphPad Software, San Diego, California USA; http://www.graphpad.com.

On average, 630 individuals (± 71.28) out of 1,000 fertilized embryos settled on each plate at the stage of postlarva, morphologically identical to an adult. Thus, a reproductive success of 0.63 was recorded. In addition, a prompt decrease of densities due to early mortality was exhibited by all treatments after 24 h from the transfer of plates in the experimental tanks. In particular, at day 1, individuals fed on Shellfish diet at low concentration exhibited a survival rate of 60.9%; individuals under Compound feed treatment exhibited a survival rate of 54.5%; individuals under M&S treatment exhibited a survival rate of 44.8% (Fig. 3A,C,E). After the first week, a larger mortality step was observed in the low concentration treatments, especially evident in the treatment Compound (Fig. 3C). At the end of the experimental period (30 days), in the low dose treatments 247 (± 2.82) individuals/plate were recorded under the Shellfish diet; 206 (± 15.33) individuals/plate under the Compound diet; and 177 (± 2.57) individuals/plate under M&S diet, corresponding to an average mortality of 66.7% referred to the individuals settled (Fig. 3A,C,E). Lower mortalities were exhibited by high concentration treatments, in the case of Shellfish and Compound diets (Fig. 3B,D). In contrast, a larger mortality event was recorded in the treatment M&S after the third week of the experiment (Fig. 3F), when most individuals exhibited swelling and bacterial films covering their surface, detected under the optical microscopy.

### Growth rates

**Figure 4:**
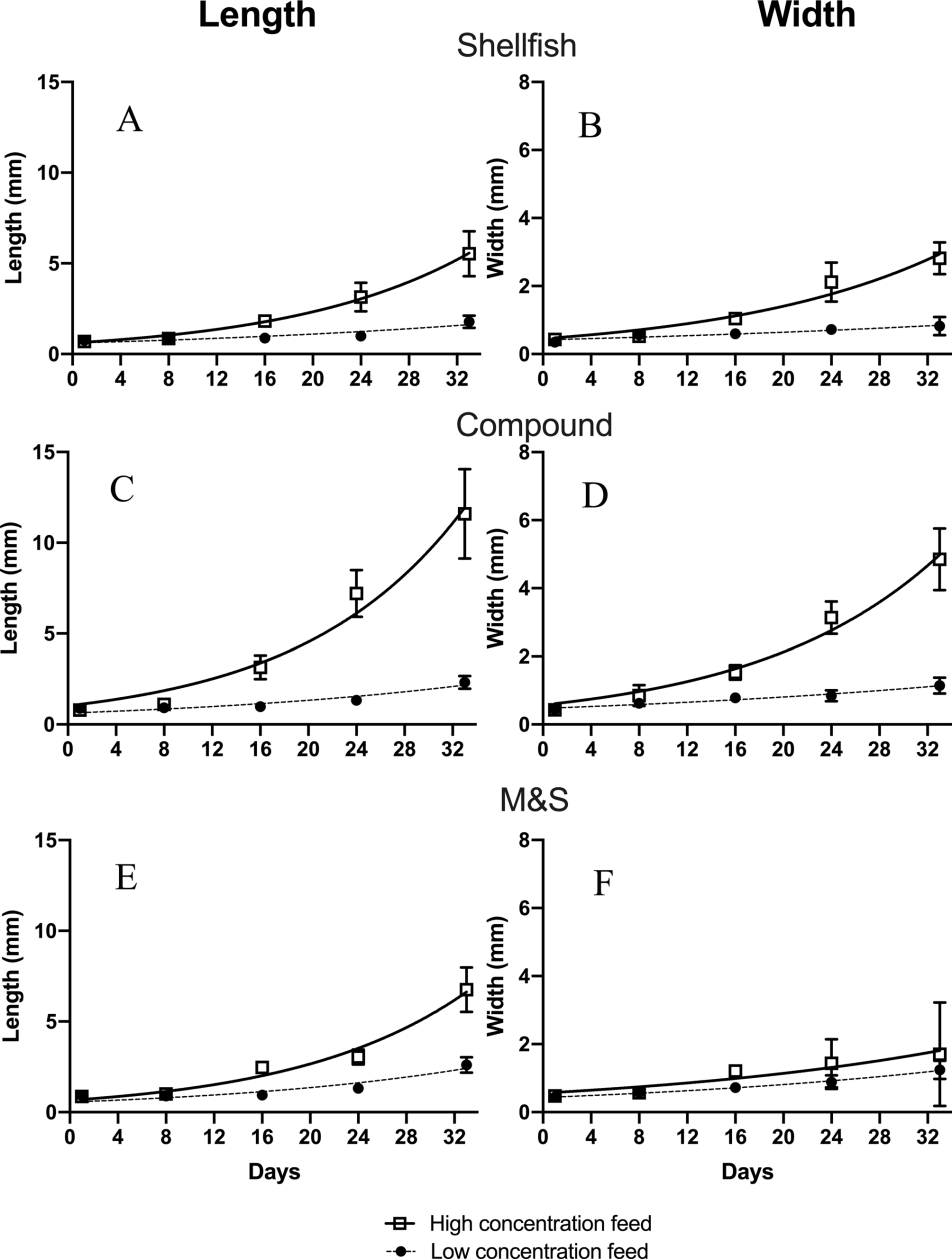
Growth of individuals according to dietetic regimes imposed, at high and low doses of feeds. (**A**,**B**) Shellfish; (**C**,**D**) Compound; (**E**,**F**) Milk&Spirulina. Figure produced using GraphPad Prism 8.0.0 for Mac, GraphPad Software, San Diego, California USA; http://www.graphpad.com.

**Figure 5:**
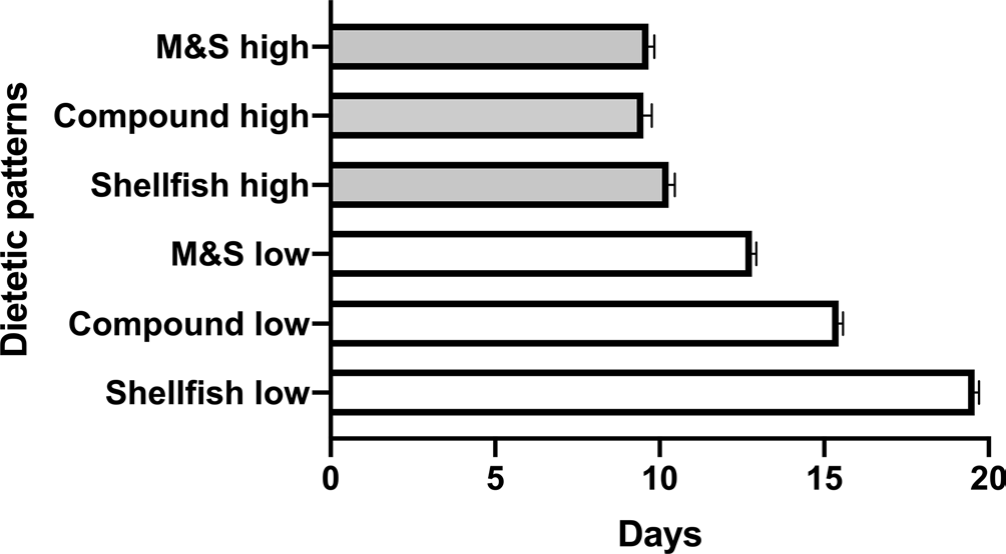
Doubling times (in days) evaluated for the length of *C. robusta* reared under the 3 diet treatments herein considered, at two concentrations (indicated as “low” and “high”). Figure produced using GraphPad Prism 8.0.0 for Mac, GraphPad Software, San Diego, California USA; http://www.graphpad.com.

Size increases of *C. robusta* consistently exhibited exponential patterns in all dietetic treatments when the Malthusian growth curve was imposed (Fig. 4). Length differences among all treatments, at both doses, were not significant during the first 2 weeks (ANOVA, p > 0.05), when the average size of individuals was consistently lower than 1 mm (Fig. 4). Clear differences emerged among treatments at high concentrations, at the end of our experimental trials (at the fourth week), with the treatment Compound producing the largest sizes (11.59 mm ± 2.46 length by 4.85 mm ± 0.90 width; Fig. 4C,D), followed by M&S (6.75 mm ± 1.2 length by 1.7 mm ± 1.5 width; Fig. 4E,F) and Shellfish diet (5.5 mm ± 1.2 length by 2.8 mm ± 0.5 width; Fig. 4A,B). In addition, lengths at the end of the experiment were significantly lower (p < 0.001) at low concentration of feeds, for all treatments. In fact, lengths reached at the end of experiments at low doses of feeds were 1.77 ± 0.3 (Shellfish), 2.3 ± 0.3 (Compound) and 2.6 ± 0.4 (M&S), respectively (Fig. 4). Thus, higher doses of feeds prompted a significantly higher growth than low doses (multiple *t* test, p < 0.001). In parallel, average widths of individuals (Shellfish 0.8 ± 0.2; Compound 1.1 ± 0.2; M&S 1.2 ± 0.2) were lower at lower doses of feeds. Length was a good descriptor of growth, since it exhibited the largest intervals of variation (Fig. 4); however, according both to length and width the treatment “Compound” prompted the highest growth rates. This result was also mirrored (Table 6) in the analysis of the Malthusian growth equations. Comparable K values were obtained according to width in all three treatments at low concentrations (Shellfish 0.02, Compound 0.02 and M&S 0.03). At high concentrations the best performance was exhibited by Compound diet, because in this case K values evaluated for width were 0.06 (Shellfish), 0.07 (Compound) and 0.04 (M&S), respectively. Similarly, when the growth constant K was evaluated on length (Table 6), low concentration treatments prompted values of 0.03 (Shellfish), 0.04 (Compound), and 0.05 (M&S), but differences were not significant (*t* test, p > 0.05) while high concentration treatments yielded K values of 0.067 (Shellfish), 0.074 (Compound) and 0.070 (M&S). Thus, M&S produced slight faster growth at low concentrations, while Compound diet promoted the best growth results at higher concentration (Table 6). This result is translated into the doubling times (time needed to double the size of individuals according to the Malthusian equation): at low density of suspended particles, Shellfish prompted the longest doubling time (about 20 days), followed by Compound feed and M&S (15 and 13 days, respectively; Fig. 5). At high doses of suspended particles all three feeds prompted a doubling time of about 10 days, but the compound feed yielded the best results, prompting a doubling time as low as 9.5 days (Fig. 5) although this result did not significantly differ from other treatments (p > 0.05, multiple *t *test).

### Molecular responses to diets at high concentration

**Figure 6:**
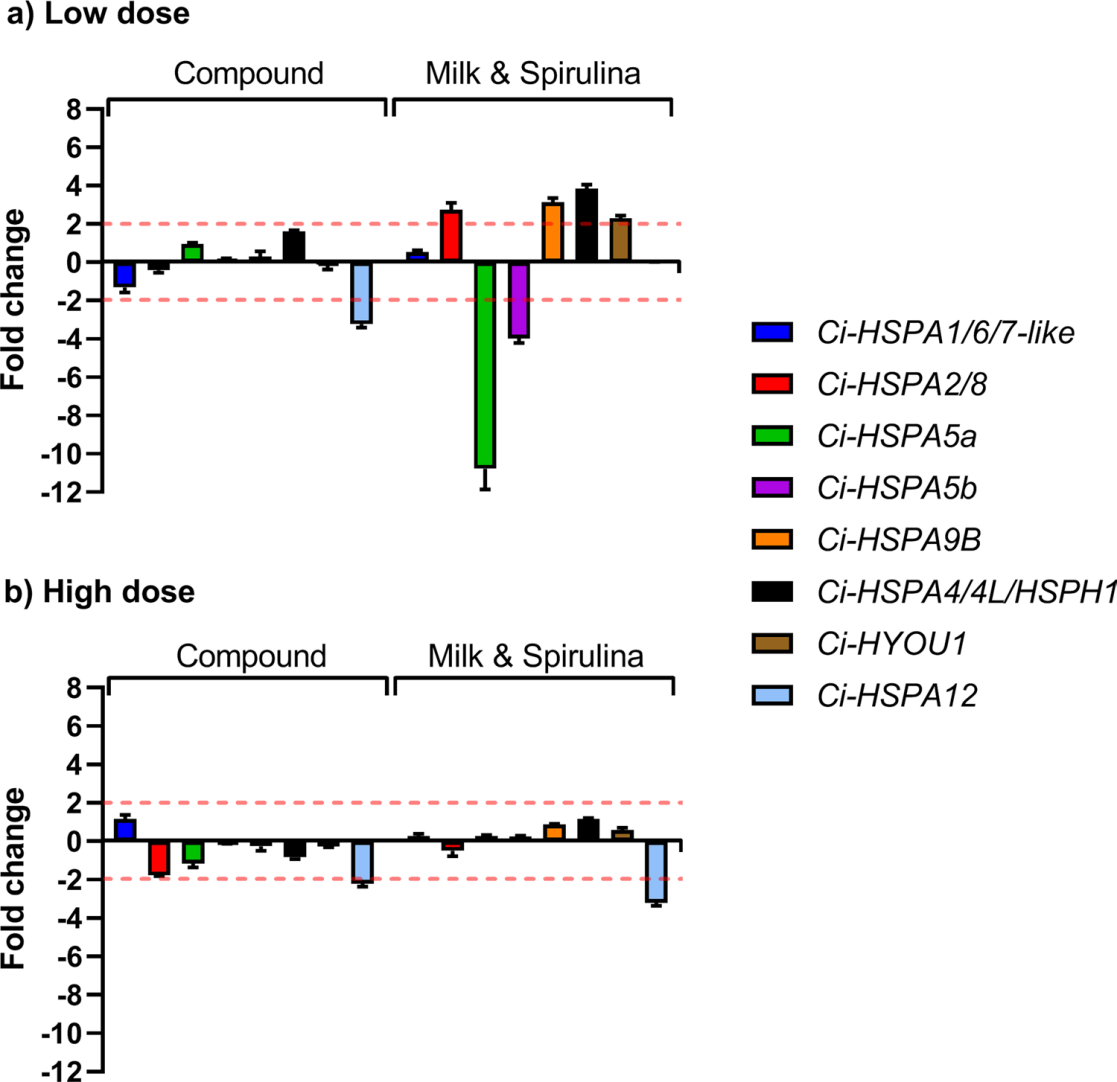
Differences in the expression levels of heat-shock proteins genes analysed in juveniles fed (**a**) at low dose and (**b**) at high dose of compound and Milk&Spirulina diets followed by qPCR. Data are reported as a fold difference compared with control, represented by juveniles fed on Shellfish (mean ± SD). Fold differences larger than ± 2 (see dotted horizontal guidelines at values of + 2 and − 2) were considered significant (see Supplementary Table S1 for the values). Figure produced using GraphPad Prism 8.0.0 for Mac, GraphPad Software, San Diego, California USA; http://www.graphpad.com.

**Figure 7:**
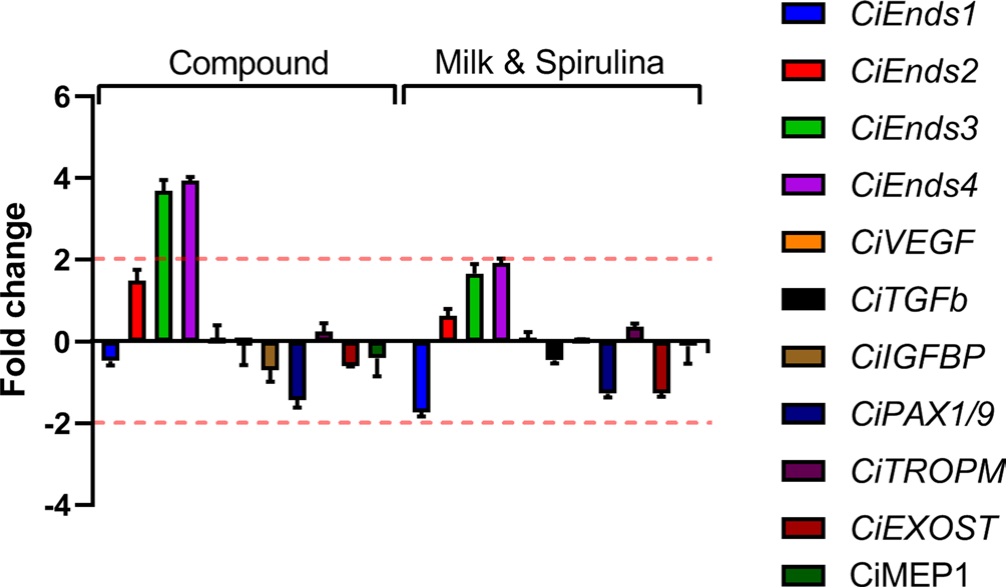
Differences in expression levels of genes involved in the formation of the endostyle and developmental processes of *C. robusta* juveniles fed at high dose of Compound and Milk&Spirulina diets followed by qPCR. Data are reported as a fold difference compared with control, represented by juveniles fed on Shellfish (mean ± SD). Fold differences larger than ± 2 (see dotted horizontal guidelines at values of + 2 and − 2) were considered significant (see Supplementary Table S1 for actual values). Figure produced using GraphPad Prism 8.0.0 for Mac, GraphPad Software, San Diego, California USA; http://www.graphpad.com.

The gene expression of juveniles fed on the Compound diet and M&S were compared with those fed on Shellfish, considered as the control diet. The analysis of gene expression of eight heat shock proteins at low feeding doses shows an increase of the expression levels of four heat-shock proteins after feeding on M&S with respect to the Shellfish diet: *Ci-HSPA2/8* (2.7), *Ci-HSPA9B* (3.1), *Ci-HSPA4/4L/HSPH1* (3.9) and *Ci-HYOU1* (2.3) (Fig. 6; Supplementary Table S1). This diet also induced a down-regulation of *Ci-HSPA5a* (− 10.8) and *Ci-HSPA5b* (− 4.0). Otherwise, the compound diet only affected the expression levels of a single gene, down-regulating *Ci-HSPA12* (− 3.2). At high feeding doses, only the gene *Ci-HSPA12* was down-regulated by both Compound diet and M&S. Moreover, we followed the expression levels of ten genes involved in the endostyle formation and in the developmental processes (Fig. 7; Supplementary Table S1) to detect morphological effects of diets on *C. robusta* development. Juveniles fed on the compound diet showed an increase of expression of two endostyle-specific genes, *Ci-Ends3* (3.7) and *Ci-Ends4* (3.9), with respect to Shellfish diet. No variations in the expression of genes involved in juvenile development were detected. Otherwise, M&S induced gene expression variation only in the gene *Ci-Ends4* (3.9).

## Discussion

Culture methods and experimental designs may fit various locations, available space, funding and manpower^26^. The experimental design herein described, set as a modification of a patented automatic rearing system for the culture of model organisms^43^, was sufficient to reduce the need for operator times, since it automatically provided water changes and feed administrations, and only required the periodical restocking of feeding reservoirs. The automatic system assured continuous control of the chemical quality of water at low concentration of feeds and when Compound feed was tested. However, it was almost unsatisfactory in the treatment M&S at higher feed concentrations, as demonstrated by the continuous increase of nitrate and phosphate during the experimental period. This might explain the episode of mortality influencing the treatment M&S after the third week of experiment. Similarly, the diet Shellfish produced sufficient growth and survival rates, especially at higher concentrations, but also led to an increase in nitrates and phosphates that the automatic system hardly controlled during the second half of the experiment. The automatic system performed at its best when feed doses were lower and produced acceptable results, at higher concentrations, in the treatment Compound, evidently promoting lower pollution of water, although most chemical and physical parameters were kept in the range preferred by this species^35^. Particle concentrations tested in this study represent sub-threshold levels for most tunicates, because negative effects of particle additions would be expected at concentration greater than 46 mg l^−1^ according to previous investigations^44^.

Survival rates and growth curves indicate animal functional responses to food concentrations, and show the performances under various dietetic regimes. Functional responses under low concentration of feeds did not differ, although the quality of water was kept in ideal conditions in these treatments. Evidently, the stress produced by insufficient feeding kept both the size and the survival of *Ciona* at minimum levels. This conclusion is also supported by records of organic particle concentrations at the end of the 3-h feeding periods^45^. In fact, suspended particles were quickly depleted at low concentration of feeds, and cultured individuals were partially starved. Due to this evident deficiency, the final size of cultured individuals differed even within low-concentration treatments. Growth constants were slightly different, showing an increasing efficiency of Shellfish vs. Compound *vs.* M&S. In these conditions (low doses of feeds), M&S maximized its efficiency, possibly due to microbial proliferation as an alternative food source, while minimizing the pollution of water, due to scarcity of food. In fact, previous authors^46^ observed that *C. robusta* exhibited a positive relationship between ingestion rate and cell concentration of *Isochrysis galbana* and that the largest filter-feeding efficiency was recorded at 5 × 10^3^ cells/mL. Similarly, investigations^44^ indicated that ingestion rates increased linearly with food concentration in another tunicate (*Halocynthia pyriformis*). However, other studies^35^ did not detect a clear maximum in the feeding rates of tunicates, according to the concentrations of particles. In our case, when cultures were managed at higher doses (ad libitum) residual suspended particles were still present before water changes, demonstrating that individuals never starved. The gut of *Ciona* reaches an intake threshold at high food concentrations, above which the clearance rate is reduced as a form of protection against gut saturation^36,41,47^. At high loads of suspended particles a reduction in ingestion rates was associated with satiation^46^ and an active rejection^47,48^ (i.e., squirting) was observed, while a reduction in lateral cilia beat frequency^47^ influenced the ingestion rates. Consequently, in conditions of high particle concentrations^48^ the best growth performances (expressed by K) were exhibited by the treatment Compound, followed by M&S and Shellfish, but in this case the differences among treatments were quite slight. In contrast, differences among treatments were significant when feeds were dosed at low concentrations, indicating that the first factor influencing growth is the concentration of feeds and that the quality of feeds^49,50^ becomes important at lower concentrations of suspended particles. In addition, M&S produced high mortality events at higher concentrations, probably due to a proliferation of microorganisms (as observed under optical microscopy, revealing the presence of abundant bacterial films and blooms of small ciliates) that negatively influenced the clearance rates and the physiology of *Ciona*^51^. Following these evidences we may conclude that, although a diet able to maximize bacterial production may be beneficial for *Ciona* at lower concentrations of particles, its deleterious effects in terms of pollution of water and triggering of opportunistic bacteria blooms may drastically reduce survival rates and growth patterns at higher concentrations, as previously observed for various ascidians^52^. Also Joly^26^ observed that artificial feeds might be scarcely suitable for juveniles, as *Ciona* failed to grow and died, due to water pollution.

Morphological data are explained by molecular investigations. In the present work, for the first time, we demonstrated that diets provided at low rates induced stress in *C. robusta* juveniles (see the Heat-map in the Supplementary Figure S1). In particular, M&S targeted the expression levels of more genes (six out of eight analysed, considered stress-inducible genes) than compound diet (one out of eight genes) but stress conditions were not detected at the highest feeding concentration. These results show that dietetic regimes are important for survival of juveniles especially when dosed at low concentrations, so explaining at the molecular level the recorded survival rates and the differences among diets. Considering this stressful condition for *C. robusta* juveniles, the expression of genes involved in the formation of the endostyle and in developmental processes was only studied in juveniles fed at high concentration. Juveniles fed at high doses of Compound diet exhibited an overexpression of two genes involved in the endostyle formation^25,53^ (an organ assisting chordates in filter-feeding), and only one of them was also targeted by M&S, even if at low rates. Also in this case, molecular data supported morphological observations, since we detected an increase in the expression levels of two endostyle-related genes in juveniles fed on the Compound diet, which produced the best growth rates.

Substantially, the feeding processes of ascidians are well established^44^ and we demonstrated that *C. robusta* follows such a general mechanism. Ideal feeds for culturing *Ciona* must fulfil some important criteria: they must be easy to obtain and to prepare, provide sufficient nutrients for growth and reproduction, and pollute as little as possible^54^. Although previous reports on the effects of feed concentration are slightly contradictory and comparisons are sometimes difficult to make, the basic principles of filter-feeding are well-known and they have been confirmed here. Differences in species, temperature, conditions (experimental diets vs. natural diets, static vs. flow-through conditions, use of weight-specific feeding measurements, wet vs. dry weight), the units used to describe the particle concentration and the use of different feeding measurements produce wide scattering among results by various authors^40^. Carver^33^ and Petersen^47^, both pointed to the variability in filtration estimations for *C. robusta*, even at similar temperature regimes. Overall, our preliminary hypotheses were confirmed, since the Shellfish feed produced a low level of survival at low doses, but it produced less than 20% of mortality after 1 month of experiment at higher doses and assured a moderate growth and it was actually the easiest and cheapest feed tested; the Compound feed containing live prey was the most effective, both for survival and growth, but it was more complex to be produced and administered. The fermented feed was simple and effective, but it demonstrated to be almost dangerous at higher concentrations, producing bacterial diseases and mortality, as revealed by microscopy observations. In fact, bacterial films covering most individuals were detected under the optical microscopy and blooms of ciliates invaded the bodies of cultured specimens. In addition, our hypotheses about doses were confirmed, taking into account previous papers indicating the feeding activity of tunicates^48^ and morphological data along with molecular investigations demonstrated that the growth constants of various feeds are comparably low at low concentration of suspended particles, because growth is fundamentally limited by starvation. However, at low concentration of feeding particles the association of a fermentable substratum (milk) with a protein-rich algal item (*Spirulina*) provides the best results in terms of growth (K = 0.031) because it maximizes the presence of food items suspended in the water. In contrast, when *C. robusta* was fed ad libitum*,* only slight differences among feeds emerged and a compound feed, containing both live particles and organic detritus, maximized both growth and survival. Such a nutritive feed assured a growth constant as high as 0.065, indicating that mature individuals suitable for experimental purposes (about 20–25 cm length), may be obtained in less than 2 months in an automatic device. In contrast, working at lower concentration of feed particles, the best results in terms of growth were obtained adopting the diet M&S (doubling time 12.81 days). In this case, to obtain mature individuals of only 6 cm length, a growth period of 3 months should be forecasted but, as demonstrated by our experimental trials, this diet must be kept at lower density of particles to avoid larger mortality due to bacterial blooms, although bacterial diseases could be further controlled using Gentamycin, according to Joly^26^. Additional dietetic adjustments could be attempted in future^55^, as a key prerequisite for the isolation, maintenance, and experimental use of stable transgenic lines^26^, taking into account the variable growth patterns prompted by different diets and the advantages provided by an automatic rearing system (along with its constraints), as shown by this investigation.

## Material and methods

This investigation aimed at defining (a) the effect of particle concentration and (b) the effect of diet composition on the growth and survival of *Ciona robusta* reared in aquarium cultures. To this end, we produced *C. robusta* larvae and post-larvae by in vitro fertilization of its gametes, and tested the effects of three diets, at two concentrations (Table 2), on their growth and survival rates.

**Table 2:** All dietetic regimes have been tested at low and high concentrations (*ad libitum*). Weights indicated in the tables (in µg dry weight of feed per litre of seawater) are referred to the concentrations obtained after each administration of feed, in each litre of culture medium at the start of the experiment (first quantity) and after 1 month of grow-out (second quantity). “Shellfish” is referred to the Shellfish diet available on the market and ready to use; “Compound” is referred to a mixed diet containing both live and dry particles (Table 3); “M&S” is a simple diet made of dry milk and dried *Spirulina* (Table 3).

	Shellfish (µg/L)	Compound (µg/L)	M&S (µg/L)
Low concentration	90–350	90–350	90–350
High concentration	350–5,000	350–5,000	350–5,000

### Collection

Adult sea squirts were collected by scuba divers on mussel culture ropes at depths between 6 and 20 m in the Ionian Sea off Taranto (Italy) in October 2018. Individuals showing no signs of damage were manually selected on site, stored in refrigerated containers and transferred to Napoli (Italy) at the Stazione Zoologica facility for the culture of model organisms. They were reared in 400 L open cycle aquariums up to the collection of gametes.

### In vitro fertilization

Mature specimens were identified according to the size of gonoducts, as observed through the transparent body: eggs and full oviducts are reddish or brownish; sperm and sperm ducts are whitish. Continuous illumination was applied until the collection of gametes, to prevent spontaneous spawning. Gametes were collected by dissecting the body wall of three individuals with a scalpel, to reach ventrally located oviducts and sperm-ducts. Eggs were collected by a Pasteur pipette and transferred into a 200 mL glass vessel for complete mixing of gamete pools. Sperm was obtained from three sperm-ducts after dissection of body walls, pooled, and diluted with 2 mL of filtered (Millipore 0.22 µm) seawater. The concentration of gamete suspensions was determined by means of a cell counting chamber. Sperm was added to pools of 1,000 eggs poured in plastic Petri dishes filled with 150 mL of filtered (Millipore 0.22 µm) seawater, in order to fertilize at a ratio of 5,000:1 of sperm to eggs, to prevent polyspermy. Subsequently, Petri dishes containing fertilized eggs were incubated for three days in a thermostatic chamber (18 °C; 12/12 photoperiod) to allow embryo development. A partial change of the culture medium was provided using filtered seawater after the complete settlement of larvae, and 150 µL of Shellfish food were added in each dish to avoid larvae starvation prior to start the feeding experiments. The density of settled larvae in each Petri was assessed at this stage, under stereomicroscopy, prior to moving culture dishes to experimental tanks (experimental time “0”).

### Experimental diets

Three diets were set and compared:

(a) **Ready-to-use commercial diet** (indicated as “Shellfish” in Table 2). The feed Shellfish (Shellfish Diet1800; Instant algae, Reed Mariculture Inc. USA), is suggested for growing-out filter feeders^56^, as a complete diet requiring minimum experimental efforts. The liquid suspension contains six marine microalgae (*Isochrisis* sp., *Pavlova lutheri*, *Tetraselmis* sp., *Chaetoceros calcitrans*, *Thalassiosira weissflogii*, *T. pseudonana*) surrounded by a dense liquid, sufficient to assure its long storage at low (4–5 °C) temperatures. It provides nutrition for bivalve species, from first feeding larvae to adult broodstock, increasing both growth rate and survival, thanks to the presence of diatoms with a high fatty acid profile^57,58^. Shellfish diet is also suggested as an excellent feed for ascidians, sea cucumber larvae, soft corals, *Artemia*, and some copepods, because it assures absence of toxic metabolites^49,50,59,60^, with respect to live cultures of microorganisms. The dry weight of Shellfish diet corresponds to 8% of its fresh weight. Cell counts in the suspension vary due to the natural origin of the product, but it roughly corresponds to 2 × 10^9^ cells mL^−1^.

(b) **Compound diet** (indicated as “Compound” in Table 2) containing both live and conserved food items (Table 3) according to various approaches followed by previous authors^56,61,62^. In line with the suggestions of various authors^26,62^ we mixed live algae (*Rhodomonas* sp. and *Isochrisis galbana*) with dry feeds (yeast, freeze-dry eggs, dried Milk, *Spirulina*) with the addition of a fat-rich algal derivative (Algamac), increasing also the levels of such unsaturated fatty acids as DHA and EPA. The actual composition of live feeds may influence the physiology of consumers at various levels^63,64^; thus, any mixture of live feeds must be produced in controlled conditions and accurately tested on target consumers.

**Table 3:** Ingredients (reported as percentage) contained in three experimental diets and their contribution in weight. “Shellfish” is referred to the Shellfish diet available on the market and ready to use, containing a mixture of 5 marine microalgae (*Isochrisis* sp., *Pavlova* sp., *Tetraselmis* sp., *Thalassiosira weissflogii* and *Thalassiosira pseudonana*); “Compound” is referred to a mixed diet containing both live and dry particles; “M&S” is a simple diet whose abbreviation is referred to Milk&Spirulina.

Ingredient	Shellfish (%)	Compound (%)	M&S (%)	Average particle size (µm)
*Microalgae mix*	100	–	–	10–600
*Dried spirulina*	–	30	50	5–70
*Milk powder*	–	10	50	10–250
*Rhodomonas *sp*.*	–	30	–	10–30
*Isochrisis *sp.	–	5	–	4–6
*Dried egg yolk*	–	20	–	5–250
*Dried yeast*	–	3	–	3–40
*Algamac*	–	2	–	100–300

(c) **Simplified compound diet** (indicated as “M&S” in Table 2) maximizing the use of milk proteins and cyanobacteria. Since Sigsgaard^61^ suggested the use of feeds able to produce bacterial proliferation (*Ciona* is able to feed on live bacteria and algae) we tested this diet made of milk powder (as a possible substrate for bacterial proliferation) and *Spirulina* (a protein-rich cyanobacterium).

The three formulations were administered at two different doses (namely, Low, 90–350 µg/L and high, 350–5,000 µg/L) in order to assess the effect of particle concentrations. These doses contained, on average, about 300 (low dose) or 8,000 (high dose) food particles per mL of culture medium.

### Experimental setup and rearing system

This experiment was conducted using a patented (E.U. Patent 102015000012043) automatic rearing system^43^ modified for the culture of *Ciona* (Figure S2). It consists of glass tanks (35 L of net volume) containing natural seawater and equipped with a computer-driven system for water changes and feed administration. Seawater, collected at 15 m depth in the Bay of Naples by an impelling system, was decanted, filtered (mechanical and activated charcoal external filters aided by skimmers) and temperature-adjusted into a large sump, then pumped from the sump at 3-h intervals, after flushing out the culture medium present in the tanks. Centrifugal pumps connected to a central control unit provided water transferrals in and out of the culture tanks. Feeds were charged into 5 L glass flasks and cyclically pumped into individual tanks by means of peristaltic pumps. Recirculating pumps were operated in each tank to assure continue re-suspension of feeds. The central operating unit was programmed in order to cyclically repeat the sequence: (1) water discharge for 10 min; (2) water charge for 10 min; (3) food addition for 15 min; (4) rearing with recirculation pumps for 3 h. Ten replicate Petri dishes (diameter 10 cm), each containing already settled *Ciona robusta*, were tested for each diet and each dose according to the experimental scheme (Table 4), for a total of 60 Petri dishes analysed. They were reared in 12 independent tanks (two tanks for each diet and each dose). Petri dishes were positioned vertically in each tank, fixed on PVC racks to reduce sedimentation over their bottoms and burial of juveniles. The main chemical and physical factors were monitored weekly (dissolved O_2_, pH, Redox potential, Temperature) along with concentrations of nutrients, (NH_4_^+^, PO_4_^3−^, NO_3_^−^, NO_2_^−^). In parallel, average length and width of post-larvae, and their density in each plate were measured under the stereomicroscopy, using a micrometric 10 × ocular. The experiment lasted 30 days, up to complete development.

**Table 4:** For each diet (Shellfish, Compound and Milk&Spirulina, whose composition is reported in Table 3), two replicate tanks were set, each containing 5 Petri dishes with *Ciona robusta*. Thus, ten replicates were managed for each treatment at low or high concentration of feeds, according to the doses reported in Table 2. Feed names are referred to the compositions indicated in Table 3.

Tank nr	Nr replicate Petri	Dose	Feed
1	5	High	Shellfish
2	5	High	Shellfish
3	5	High	Compound
4	5	High	Compound
5	5	High	M&S
6	5	High	M&S
7	5	Low	Shellfish
8	5	Low	Shellfish
9	5	Low	Compound
10	5	Low	Compound
11	5	Low	M&S
12	5	Low	M&S

### Collection, RNA extraction and gene expression by qPCR

Fifty specimens were collected at the start of the experiment and after 30 days of growth under the diet treatments at high doses. Total RNA extraction was performed using Aurum Total RNA Mini Kit (Bio-Rad), according to Ruocco^65^ and 1 μg of total RNA for each sample was retro-transcribed with an iScript cDNA Synthesis kit (Bio-Rad, Milan, Italy). The complete coding sequences of ten genes were retrieved from NCBI (available at https://www.ncbi.nlm.nih.gov/) and Joint Genome Institute (JGI) Genome Portal (https://mycocosm.jgi.doe.gov/Cioin2/Cioin2.home.html). Specific primers were designed on the nucleotide sequences, and selected fragments (Table 5) were then amplified and purified from agarose gel by the QI Aquick Gel Extraction kit (Qiagen, Milan, Italy) using a Taq High Fidelity PCR System (Roche, Milan, Italy). The specificity of PCR products was checked by DNA sequencing and then verified by melting curve analysis using qPCR. Standard curves (according to the equation E = 10^−1^/slope) were used to calculate the efficiency of primer pairs, determining Ct values *vs*. the logarithm of each dilution factor, by testing five serial dilutions (GraphPad Prism 8.0.0 for Mac, GraphPad Software, San Diego, California USA; http://www.graphpad.com). PCR efficiencies were calculated for control and target genes. Diluted cDNA was used as a template in a reaction containing a final concentration of 0.3 mM for each primer and 1 × Fast Start SYBR Green master mix (total volume of 10 μL) (Applied Biosystems, Monza, Italy). PCR amplifications were performed in a ViiATM7 Real Time PCR System (Applied Biosystems, Monza, Italy) thermal cycler using the following thermal profile: 95 °C for 10 min, one cycle for cDNA denaturation; 95 °C for 15 s and 60 °C for 1 min, 40 cycles for amplification; 72 °C for 5 min, one cycle for final elongation; one cycle for melting curve analysis (from 60 to 95 °C) to verify the presence of a single product. Each assay included control for each primer pair with no-template. Primers for the eight genes for HSP70 superfamily proteins were those reported in Fujikawa^66^. All qPCR reactions were carried out in triplicate to evaluate intra-assay variability. Fluorescence was measured using ViiATM7 software Version 1.0 (Applied Biosystems, Monza, Italy, https://www.thermofisher.com/it/en/home.html). The expression levels of each gene were internally normalized against the housekeeping gene*Ci-CA6*, which encodes cytoplasmic actin^66^, using REST software (Relative Expression Software Tool, Version 1.0, Weihenstephan, Germany. https://www.gene-quantification.de/rest.html) based on the Pfaffl method^67,68^. Relative expression ratios above 2.0 cycles were considered significant when comparing M&S and Compound diets versus Shellfish diet. Experiments were repeated at least twice, in order to obtain an experimental replicate of each qPCR plate. All used software was not freely available (Table 6).

**Table 5:** Gene name, acronym, accession numbers, primer sequences and lengths of PCR amplified fragments are reported for the genes here analysed.

Gene Name	Acronym	Accession Name	Primer	Sequence (5′ → 3′)	Amplicon length (bp)
*Paired box 1/9*	*PAX1/9*	AB020762.1	Ci_Pax1/9_F1	CTGGTATATTTGCATGGGAGATC	203
			Ci_Pax1/9_R1	CATGTTAGTGCTTTGTGGTGG	
*Tropomyosin-like protein*	*TROPM1*	AB036846.1	Ci_tropm1_F1	CACCGATATTGAGCGGTTAG	163
			Ci_tropm1_R1	GAGTTTTAGCTCCTCAGTTAG	
*Endostyle-specific gene 1*	*ENDS1*	AB010895.1	Ci_Ends1_F1	GAGGAACTCGATAAGAAGAGA	210
			Ci_Ends1_R1	CATATGGCAGGCGACGTAGTTC	
*Endostyle-specific gene 3*	*ENDS3*	AB010897.1	Ci_Ends3_F2	GCCATGCCAACCATGCATAAG	184
			Ci_Ends3_R2	CGACGACCATGTCTTCCATTG	
*Endostyle-specific gene sp1*	*ENDS sp1*	AB010896.1	Ci_Ends-sp1_F1	CTACTGAAGGAATCGCTGCTC	132
			Ci_Ends-sp1_R1	GGCATAGTATCGAGTGATGTTC	
*Endostyle-specific gene sp2*	*ENDS sp2*	NM_001032630.1	Ci_Ends-sp2_F1	CGAGATTCTTGATGGGATCAC	203
			Ci_Ends-sp2_R1	GGATACATGGTTGTCGGATAC	
*Vascular endothelial growth factor A-A-like*	*VEGF*	XM_002127976.5	Ci_VEGF_F1	CACAACGTAACCGCGTACTAC	151
			Ci_VEGF_R1	CTGCTGCTTACGACATTGGC	
*Exostosin-like gene*	*EXOST*	Cioin2|217147	Ci_Exost_F1	GACTACAGCACAGCTATAGAG	152
			Ci_Exost_R1	CCATCAGTTTCCTGCTGCAG	
*Transforming growth factor beta*	*TGFb*	Cioin2|379299	Ci_TGFb_F1	GACCTACTACGCAGAAGATG	156
			Ci_TGFb_R1	GTTGCCGACACTATTGAGTC	
*Insulin-like growth factor binding protein*	*IGFBP*	Cioin2|224443	Ci_IGFBP_F1	CTGCGGGTGCTGCATGATGTG	190
			Ci_IGFBP_R1	GACAACTGCATCGTAAACGCG

**Table 6:** Parameters of the Malthusian growth Eq. (1) imposed: y = Y_0_ * exp (K * X), where Y_0_ indicates the Y value at time zero and K is the growth constant. In this equation, doubling-time is expressed in days, and it is computed as ln(2)/K. The names of dietetic treatments are referred to the feeds indicated in Table 3.

Dietetic treatment	According to width		According to length	
	Y_0_	K	Y_0_	K
Shellfish diet low concentration	0.4185	0.0214	0.6081	0.0297
Compound low concentration	0.4754	0.0265	0.6280	0.0376
M&S low concentration	0.4324	0.3155	0.5575	0.0445
Shellfish diet high concentration	0.4525	0.0567	0.6094	0.0670
Compound high concentration	0.5738	0.0655	0.0280	0.0743
M&S high concentration	0.5611	0.0355	0.6547	0.0701

### Statistical treatment of data

Growth curves of *C. robusta* in each treatment were obtained according to the Malthusian model^69^:

1 $${\text{Y }} = {\text{ Y}}_{0} *{\text{ Exp }}\left( {{\text{K }}*{\text{ X}}} \right)$$

where Y_0_ is the size at time 0 and K is the Malthusian growth rate of the population^70^. Growth curves were compared by means of the Friedman test and the Dunn’s multiple comparison post hoc tests in order to evaluate the differences among treatments and doses. Chemical measures and survival data were tested for their normality using a Shapiro–Wilk normality test and, when normality was confirmed, the significance of differences among treatments was assessed by means of one-way ANOVA followed by Tukey’s post hoc tests. When normality was not assured, Mann–Whitney test was applied. Paired *t* tests were applied to evaluate the significance of differences in growth descriptors, between couples of dietetic treatments. All statistical analyses and graphs were performed using GraphPad Prism 8.0.0 for Mac, GraphPad Software, San Diego, California USA (http://www.graphpad.com), not freely available.

## Supplementary information

Supplementary information
