# Supplementary material for: Coupling feeding activity, growth rates and molecular data shows dietetic needs of Ciona robusta (Ascidiacea, Phlebobranchia) in automatic culture plants
Source: Sci Rep. 2020 Jul 9;10:11295. doi: 10.1038/s41598-020-68031-0 (PMC7347631; doi:10.1038/s41598-020-68031-0)
Supplement: Supplementary file 1 — Supplementary information [file 41598_2020_68031_MOESM1_ESM.pdf]

Supplementary Material

Coupling feeding activity, growth rates and molecular data shows dietetic needs of *Ciona robusta* (Ascidiacea, Phlebobranchia) in automatic culture plants

Valerio Zupo[1*], Sebastiano Scibelli[1,2], Mirko Mutalipassi[1], Nadia Ruocco[1], Francesco Esposito[1], Alberto Macina[3], Gianluca Polese[2], Anna Di Cosmo[2], Maria Costantini[1*]

[1]Department of Marine Biotechnology, Stazione Zoologica Anton Dohrn.Villa Comunale, 80121 Napoli, Italia.

[2] Università degli Studi di Napoli Federico II. Monte Sant'Angelo. 80100 Napoli. Italia

[3] Department of Research Infrastructures for Marine Biological Resources, Marine Organisms Core Facility, Stazione Zoologica Anton Dohrn, Villa Comunale, 80121 Napoli, Italia

* Corresponding author email: valerio.zupo@szn.it; mcosta@szn.it

Supplementary Table S1. Fold changes reported for each gene analysed by Real Time qPCR. Up-regulated genes = red, down-regulated genes= blue.

	Low dose		High dose	
**Genes**	**Compound**	**Milk & Spirulina**	**Compound**	**Milk & Spirulina**
Ci-HSPA1/6/7-like	-1.31	0.54	1.15	0.28
Ci-HSPA2/8	-0.42	2.74	-1.77	-0.50
Ci-HSPA5a	0.96	-10.77	-1.16	0.27
Ci-HSPA5b	0.16	-3.97	-0.06	0.24
Ci-HSPA9B	0.32	3.15	-0.27	0.88
Ci-HSPA4/4L/HSPH1	1.63	3.86	-0.83	1.18
Ci-HYOU1	-0.11	2.30	-0.27	0.59
Ci-HSPA12	-3.23	0.01	-2.21	-3.21

	High dose	
**Genes**	**Compound**	**Milk & Spirulina**
CiEnds1	-0.47	-1.73
CiEnds2	1.49	0.63
CiEnds3	3.69	1.66
CiEnds4	3.94	2.00
CiVEGF	0.05	0.10
CiTGFb	0.00	-0.45
CiIGFBP	-0.70	0.04
CiPAX1/9	-1.43	-1.27
CiTROPM	0.25	0.36
CiEXOST	-0.59	-1.26
CiMEP1	-0.41	-0.11

Supplementary Figure S1. Heat map (Heatmapper, available at the site www.heatmappear.ca) of differentially expressed genes involved in *C. robusta* endostyle formation and developmental processes. Color code: red, positive values of gene expression (up-regulated genes); green, negative values of gene expression (down-regulated genes); black, genes for which there was no variation of expression.

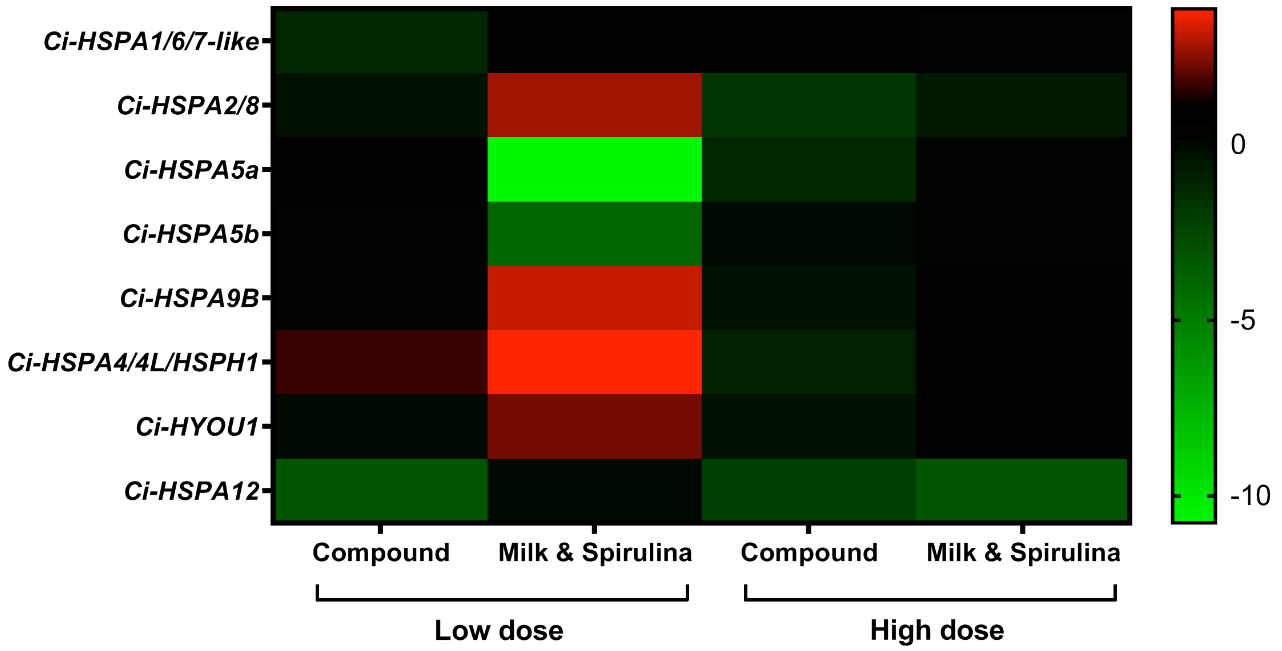

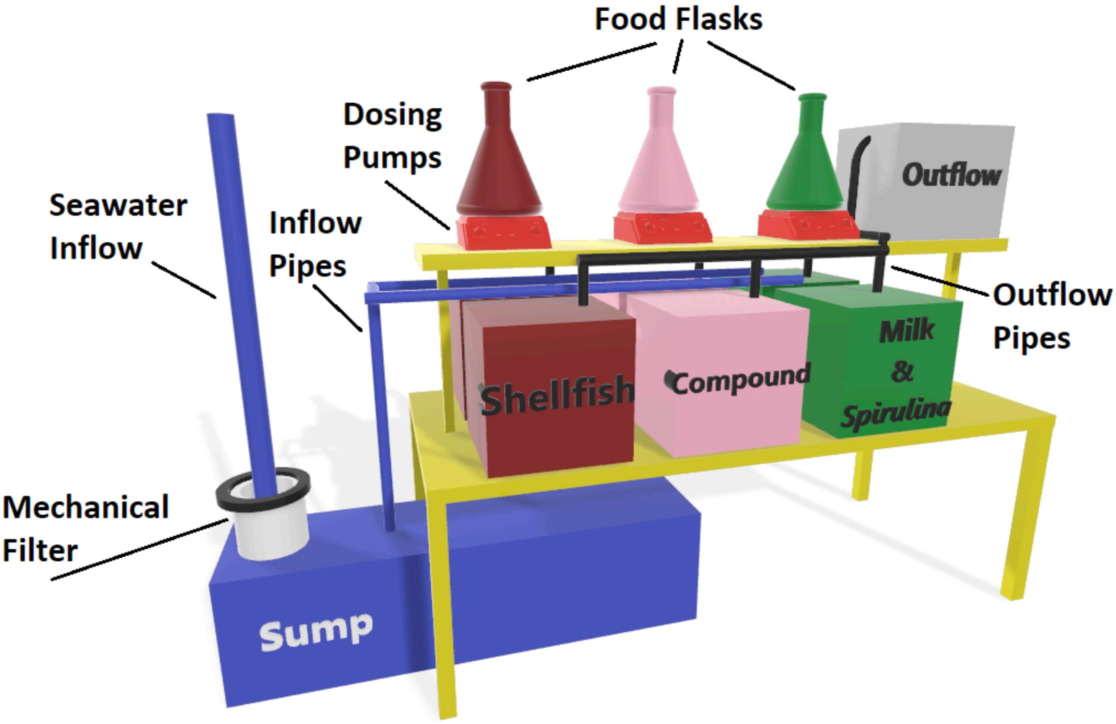

Figure S2: Experimental set-up taking advantage of an automatic device for the culture of model organisms herein described.
